# The impact of social isolation on HPA axis function, anxiety-like behaviors, and ethanol drinking

**DOI:** 10.3389/fnint.2013.00102

**Published:** 2014-01-02

**Authors:** Tracy R. Butler, Olusegun J. Ariwodola, Jeffrey L. Weiner

**Affiliations:** Department of Physiology and Pharmacology, Wake Forest University School of MedicineWinston-Salem, NC, USA

**Keywords:** hypothalamic–pituitary–adrenal axis, stress, alcoholism, dependence, basolateral amygdala, social isolation

## Abstract

Dysregulation of the hypothalamic–pituitary–adrenal (HPA) axis is often observed in alcoholics and humans subjected to early life stress, and animal models of ethanol (EtOH) dependence. We examined HPA axis function in a rodent model of early life stress that engenders increases in behavioral and neurobiological risk factors of alcoholism. Long-Evans male rats were group housed (GH) or socially isolated (SI) for 6 weeks during adolescence. We examined the corticosterone (CORT) response to stress with and without dexamethasone (DEX) and anxiety-like behaviors. Following the DEX suppression test and behavioral assays, half of the cohort engaged in 6 weeks of EtOH drinking in a homecage, two-bottle choice intermittent access model. A subset of the cohort was not exposed to EtOH, but was used for electrophysiological measurement of glutamatergic synaptic plasticity in the basolateral amygdala (BLA). Correlational analyses examined relationships between measures of CORT, anxiety-like behaviors, and EtOH intake/preference. With DEX pre-treatment, SI rats failed to suppress CORT in response to an acute stress; GH rats showed a significant suppression. In SI rats, there was a significant negative correlation between baseline CORT and elevated plus maze open arm time, as well as significant positive correlations between baseline CORT and both EtOH intake and preference. No significant relationships between baseline CORT and behavioral measures were observed in GH rats. Glutamatergic plasticity in the BLA was similar in magnitude between GH and SI rats, and was not altered by exogenous application of CORT. These data suggest that HPA axis function is affected by SI, and this is related to antecedent anxiety-like behavior and may predispose for future EtOH self-administration. Relationships between HPA axis function, anxiety, and EtOH measures in SI rats further strengthens the utility of this paradigm in modeling vulnerability for affective disorders and alcoholism.

## INTRODUCTION

Early life stress is correlated with both hypothalamic–pituitary–adrenal (HPA) axis dysfunction and alcoholism in humans (Lovallo, 2012). Further, HPA axis dysfunction has been suggested to precede the development of affective or addictive disorders (Moss et al., 1995; Lovallo, 2012). HPA axis dysfunction may include reduced or increased responsiveness to stressors as measured by circulating levels of corticosterone (CORT) and impaired dexamethasone (DEX) suppression of CORT, both of which have been noted in dependent individuals and individuals at-risk for ethanol (EtOH) dependence. Zimmermann et al. (2004) showed differential Adrenocorticotropic hormone (ACTH) and cortisol responses to social stress in healthy individuals with a positive family history for alcoholism compared to family history negative healthy individuals, thereby supporting the idea that pre-existing HPA axis reactivity may represent a biological risk factor in at-risk individuals. HPA axis dysfunction is also often an important consequence of alcoholism. For instance, abstinent male alcoholics show a blunted response to an acute pharmacological stressor at several levels of HPA axis function (Costa et al., 1996; Adinoff et al., 1998), and this blunting has been shown to be predictive of relapse (Junghanns et al., 2003). Whether HPA axis dysfunction precedes or is a consequence of EtOH dependence, pharmacological reduction of stress, anxiety, and negative affect reduces EtOH craving in alcohol dependent individuals, suggesting that normalizing function of the HPA axis and/or reducing stress/anxiety may be efficacious treatment targets for EtOH dependence/relapse (Breese et al., 2011; Fox et al., 2011). Measures of HPA axis function/reactivity can show marked individual variability. However, Besemer et al. (2011) reported that failure to suppress cortisol levels on the DEX suppression test (DST) and increased urinary cortisol are among the most consistent measures of HPA axis dysfunction reported in alcohol-induced Cushing syndrome.

Several studies have shown that early life stress in humans engenders reduced reactivity to stressors (Lovallo, 2012; Lovallo et al., 2012). The model used in the current studies imparts early life stress in rodents by taking advantage of the importance of social interaction during adolescence by comparing rats raised in groups of four (group housed, GH) to rats raised in isolation for 6 weeks (socially isolated, SI). Markedly greater anxiety-like behavior and EtOH intake has been observed in SI rats relative to GH rats (McCool and Chappell, 2009; Chappell et al., 2013), with the difference in anxiety-like behavior persisting for as many as 8 weeks along with dysregulated dopamine kinetics in the nucleus accumbens (Yorgason et al., 2013). One previous study assessed HPA axis function in male Sprague–Dawley rats that were GH or SI for 30 days and showed that SI rats were also less effective in suppressing CORT in response to DEX than GH rats (Serra et al., 2005).

Corticosterone -related plasticity has been observed in several brain regions including the basolateral amygdala (BLA), which plays a major role in the regulation of anxiety, stress, and reward. BLA activation is associated with an increase in anxiety-like behaviors in rodents, and humans with stress and/or affective disorders show increased basal amygdalar activity (Drevets, 1999). Plasticity in the BLA following chronic stress *in vivo* is correlated with increased anxiety-like behaviors, dendritic hypertrophy, and increased spine density. These changes also correlate with enduring increases in anxiety-like behavior, at least 3 weeks post-stress (Vyas et al., 2002, 2004; Vyas and Chattarji, 2004; Mitra et al., 2005). In hippocampal neurons, CORT/stress has been shown to influence pre- and post-synaptic mechanisms of glutamatergic plasticity (Tse et al., 2012). However, CORT has opposite effects on mEPSC frequency in CA1 pyramidal neurons and BLA pyramidal neurons from stress naïve rats (Karst et al., 2010), and Pu et al. (2009) showed that long-term CORT occluded, whereas short-term CORT facilitated, LTP in BLA neurons. *In vivo*, blockade of the low-affinity CORT receptor (glucocorticoid receptor; GR) has also been shown to be quite important in EtOH-related phenomena, as the GR antagonist mifepristone has been shown to: (1) block stress-induced reinstatement of drinking (Simms et al., 2012); (2) block the development and maintenance of EtOH dependence (Vendruscolo et al., 2012); (3) block voluntary EtOH intake in a limited access procedure (Koenig and Olive, 2004); (4) block somatic signs of EtOH withdrawal (Sharrett-Field et al., 2013); and (5) block EtOH withdrawal anxiety-like behavior (Jacquot et al., 2008). Together, these data emphasize a need for investigation into mechanisms of plasticity in the BLA related to CORT signaling and behavior.

Although a relationship between anxiety and addiction is well-accepted, with a comorbid diagnosis increasing the risk of alcohol relapse (Driessen et al., 2001), the contribution of HPA axis dysregulation and CORT following early life stress to the development and maintenance of neuroplasticity that contributes to increases in anxiety-like behaviors and EtOH intake are unclear. Behavioral and neurobiological effects of CORT have been modeled using many different paradigms, and what appears clear is that effects of stress or CORT depend largely on the type and length of stressor or CORT exposure, and the brain region being studied. To more clearly understand the relationship between long-term stress as it relates to altered CORT signaling and alcoholism, we employed a model that engenders a behavioral phenotype in male Long Evans rats that recapitulates many important attributes of alcoholism. The current study sought to replicate previous results from our lab showing greater anxiety-like behavior and EtOH intake in male Long Evans rats, as well as to determine whether this model of chronic adolescent stress also engenders HPA axis dysregulation. We also assessed relationships between HPA axis function and anxiety-like behaviors, EtOH intake/preference, and BLA glutamatergic plasticity. We hypothesized that HPA axis dysregulation would be apparent in SI rats, and that measures of HPA axis function would be correlated with anxiety-like behavior and EtOH intake/preference.

## MATERIALS AND METHODS

### SUBJECTS

Male Long Evans rats (*N* = 34) were used in these studies (Harlan Laboratories, Indianapolis, IN, USA). Rats arrived immediately post-weaning on post-natal day 21 (PND 21), and were GH (4–5/cage; 33.0 cm × 59.7 cm; Nalgene, Rochester, NY, USA) for 1 week. At PND 28, rats were either maintained as GH (4/cage, *n* = 16), or were SI in a standard laboratory cage (SI; 25.4 cm × 45.7 cm, *n* = 18) for 6 weeks with minimal handling (one cage change/weighing per week). GH and SI rats remained in their housing conditions until the completion of behavioral tests, but were single housed for homecage EtOH self-administration. Rats were always maintained in the same colony room. All animal care procedures were in accordance with the NIH Guide for the Care and Use of Laboratory Animals (NIH Publications No. 8023) and approved by the Institutional Animal Care and Use Committee.

### BEHAVIORAL TESTING

**FIGURE 1:**
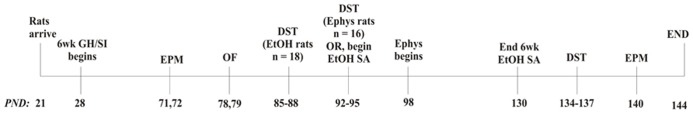
**Experimental design.** DST, dexamethasone suppression test; Ephys, electrophysiology; EtOH, ethanol; GH, group housed; EPM, elevated plus maze; OF, open field; PND, post-natal day; SA, self-administration; SI, socially isolated.

A timeline of the complete experimental design is included (**Figure 1**). Following the 6 week housing procedure, behavioral tests were conducted over a 4 week period. At PND 71–72, anxiety-like behavior was measured using the elevated plus maze (EPM; Med Associates, St. Albans, VT, USA). Dependent measures included time spent in the open arms, number of open arm entries, number of open arm explorations, and number of closed arm entries. At PND 78–79, locomotor behavior in a novel environment was measured using open field locomotor activity chambers (Med Associates). Dependent measures included total distance traveled, vertical time, center time, and margin time. Anxiety-like behavior and locomotion were tested between 8:30 and 10:30 a.m. At PND 85–88 or 92–95, HPA axis responsivity to a stressor and negative feedback were measured, respectively, following swim stress and administration of either vehicle (VEH; 0.01% propylene glycol (PG) in saline, s.c.) or DEX (50 μg/kg, s.c.), in which the dependent variable was level of plasma CORT. This procedure is detailed below. After these procedures, half of the cohort (*n* = 18) began 6 week EtOH self-administration (detailed below) and the other half of the cohort was used for electrophysiology studies (*n* = 16; detailed below). The rats that self-administered EtOH were re-tested on the EPM and DST at PND 134–140 (**Figure 1**). Previous data have shown that 28 days between testing on the EPM is sufficient for reliable re-testing, suggesting that when allowing sufficient time between tests, the EPM is a measure of trait, not state, anxiety-like behavior (Schneider et al., 2011).

### DEXAMETHASONE SUPPRESSION TEST PROCEDURE

The DST was conducted with each rat on two consecutive days using a within-subject, counterbalanced design to measure stress responsivity (CORT response to a stressor with VEH pre-treatment) and HPA axis negative feedback (CORT response to a stressor with DEX pre-treatment). In healthy subjects, administration of the synthetic glucocorticoid DEX initiates engagement of a negative feedback loop for HPA axis function, thus reducing levels of circulating CORT (Cole et al., 2000). Each day, 8–10 rats were tested, but run in two groups of four to maintain strict adherence to timing of blood sampling and DEX/VEH injection. Tail bloods were taken at baseline (~8:30 a.m. for group1; ~9:30 a.m. for group2), followed immediately by injections [s.c.; 50 μg/kg DEX in PG, diluted in sterile saline to a final concentration of 0.01% PG (VEH)]. This dose of DEX was previously shown to significantly suppress the CORT response to restraint stress by 80% in rats (Cole et al., 2000). Following injection, rats were returned to their homecage for 90 min. After 90 min, rats were subjected to 5 min swim stress, as preliminary data showed that this stressor significantly increased plasma CORT levels relative to baseline (data not shown). To swim, rats were placed into an opaque polypropylene cylinder with room temperature water approximately 8 in (~20 cm) deep. Tail blood was taken again 30 min after removal from the water (post-stress). One day before the DST, tail blood samples were taken from each rat to acclimate the rats to the tail bleeding procedure. Tail bloods were immediately centrifuged and plasma was stored at -80°C until the CORT assay. The rats run during the first week of DEX suppression (*n* = 18) began EtOH self-administration the following week (at PND 91), while the remaining rats (*n* = 16) completed the DST and were then used for electrophysiology studies (detailed below).

### CORTICOSTERONE ASSAY

Corticosterone was measured from plasma obtained from tail bloods in a competitive enzyme immunoassay containing a polyclonal CORT antibody (Immunodiagnostic Systems; Scottsdale, AZ, USA). In this assay, CORT from the samples, calibrators, and controls competes with enzyme-labeled CORT for binding in a 96-well plate. The enzyme-labeled CORT reacts with a chromogenic substrate, creating color that is inversely proportional to the CORT concentration of the samples. Color intensity was detected using a microplate reader (Molecular Devices, Spectra-Max Plus384, Sunnyvale, CA, USA), with absorbance measured at 450 nm. CORT concentrations were derived from a standard curve fit to a four parametric logistic equation. This assay required a 1:20 dilution of plasma samples.

### ETHANOL DRINKING PROCEDURE

Ethanol self-administration was conducted using an intermittent access, two-bottle choice design [*n* = 18; (Wise, 1973; Chappell and Weiner, 2008; Simms et al., 2008)], during which time all rats were single housed and remained so for the duration of the study. In this model, rodents were given two bottles in their home cage containing 20% EtOH and water, respectively, on Mondays, Wednesdays, and Fridays. Water and EtOH consumption were measured after 30 min and 24 h (daily) access to EtOH. An EtOH preference ratio (EtOH drank/total fluid intake) was calculated at each time point. Data from our lab have previously shown that BECs at the 30 min time point in this model (in rats showing similar intake levels) approximate 40 mg/dl (Chappell et al., 2013). Water and EtOH were given in graduated drinking tubes (Med Associates), and the position of the bottles was alternated on each drinking day to control for potential side preferences. Rats were maintained on this schedule for 6 weeks. Rats were given *ad libitum* access to food throughout the drinking paradigm and were weighed on each drinking day.

### ELECTROPHYSIOLOGY METHODS

Following the DST, electrophysiology was conducted over the course of 4 weeks (*n* = 8 GH and *n* = 8 SI) with the experimenter blind to the experimental group. Transverse amygdala slices (400 μm) were prepared and slices were maintained at ambient temperature for at least 1 h in oxygenated artificial cerebrospinal fluid (aCSF) containing: 124 mM NaCl, 3.3 mM KCl, 2.4 mM MgCl_2_, 2.5 mM CaCl_2_, 1.2 mM KH_2_PO_4_, 10 mM D-glucose, and 25 mM NaHCO_3_, saturated with 95% O_2_ and 5% CO_2_ (Silberman et al., 2012; Rau et al., 2013). Slices were placed in the recording chamber and were continuously superfused in aCSF (2 ml/min) warmed to approximately 30°C. Electrodes were filled with an extracellular pipette solution containing 150 mM NaCl. Glutamatergic field excitatory postsynaptic potentials (fEPSPs) in the presence of bicuculline (20 μM) were elicited every 20 s in the BLA in response to stimulation of the external capsule with a bipolar stimulating electrode (FHC, Bowdoinham, ME, USA). The stimulus intensity that evoked 50% maximal amplitude was used. Following establishment of a stable baseline, CORT (100 nM) or VEH (0.01% DMSO in water) was applied for 20 min; then, in the presence of CORT or VEH, low-frequency stimulation (LFS) of the external capsule was applied (1 Hz, 15 min). Plasticity was measured post-LFS for 60 min. Amplitude of fEPSPs was converted to %control of baseline. For analyses of fEPSP amplitude, baseline was computed as an average of the sweeps for last 5 min of the baseline period; data for the drug application period was averaged across 20 min; and post-LFS plasticity was quantified as the last 30 min post-LFS. The unit of determination for these experiments was a slice, with two separate slices per rat used to measure plasticity in response to LFS with and without CORT exposure to assess whether group differences existed in response to LFS in general or if altered CORT signaling could be detected with exogenous application of CORT before LFS. fEPSP recordings were acquired with an Axoclamp 2B amplifier (Axon Instruments, Foster City, CA, USA) in the current-clamp mode and digitized (Digidata 1200B; Axon Instruments) and analyzed on- and off-line using pClamp 10.1 software (Molecular Devices).

### DATA ANALYSIS

For dependent measures of anxiety-like behavior, GH and SI data were analyzed using one-tailed *t*-tests. Activity in the open field chamber was analyzed using a repeated measures two-way ANOVA (group × time), with Bonferroni correction. For comparison of baseline CORT levels, an average of the two baseline CORT levels was computed for each rat and GH and SI rats were compared using a one-tailed *t*-test. The CORT response to stress and the effect of DEX on CORT levels in the DST were analyzed using two-way ANOVA (group × treatment), followed by one-tailed *t*-tests within groups given our a priori hypothesis of HPA axis dysfunction in SI rats. Correlational analyses were also conducted for select dependent variables using Pearson’s *r*. Electrophysiology data were analyzed using a three-way ANOVA (group × treatment × time) and Student–Newman–Kewls *post hoc* tests.

## RESULTS

### ANXIETY-LIKE BEHAVIOR AND OPEN FIELD LOCOMOTOR ACTIVITY

**FIGURE 2:**
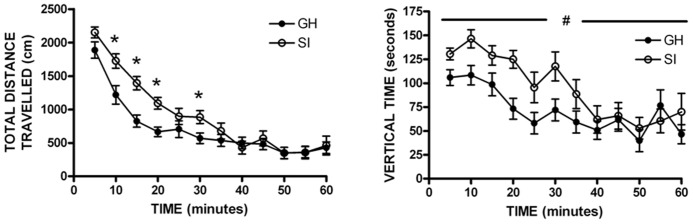
**Open field locomotor activity.** Socially isolated (SI) rats showed significantly more locomotor activity [distance traveled; group × time *F*(11,407) = 2.955, *p < 0.001] and spent more time exploring vertically than group housed (GH) rats [main effect of group, *F*(1,407) = 4.949, #*p* < 0.05]. *p < 0.05 vs. GH rats at that timepoint; #SI vs. GH rats.

Previous data from our lab and others have demonstrated that SI rats show significantly greater anxiety-like behavior than GH rats after the 6 week housing manipulation (McCool and Chappell, 2009; Chappell et al., 2013). Within this cohort, a non-significant trend was observed for SI rats to show greater anxiety-like behavior than GH rats, without a group difference in level of locomotor activity as indicated by the number of closed arm entries (**Table 1**). In regard to locomotor activity in a novel open field environment, SI rats covered significantly more distance (cm) than GH rats at several time points [group × time *F*(11,407) = 2.955, *p* < 0.001], and spent significantly more time exploring vertically [main effect of group, *F*(1,407) = 4.949, *p* < 0.05] during the 60 min test session (**Figure 2**). There was also a statistically significant interaction for time spent in the center of the open field, [*F*(11,407) = 1.951, *p* < 0.05], though *post hoc* tests indicated that this effect was driven by time, as no difference between groups was detected at any individual time point (data not shown).

**Table 1:** Group housed (GH) and socially isolated (SI) rats were tested for anxiety-like behaviors on the elevated plus maze after the 6 week housing period.

	**Group housed, GH**	**Socially isolated, SI**	***t*, *p***
Open arm time (sec)	68.82 ± 11.8	54.32 ± 11.9	*t* = 0.861, *p* = 0.198
Open arm entries	3.63 ± 0.6	2.56 ± 0.5	*t* = 1.358,*p* = 0.092
Open arm explorations	10.50 ± 1.6	8.56 ± 1.2	*t* = 0.993, *p* = 0.164
Closed arm entries	6.31 ± 0.4	6.33 ± 0.5	*t* = 0.034, *p* = 0.487

*SI rats showed a non-significant trend for greater anxiety-like behavior than GH rats. Data were analyzed using a one-tailed *t*-test and are presented as mean ± SEM.*

### DEXAMETHASONE SUPPRESSION TEST

**FIGURE 3:**
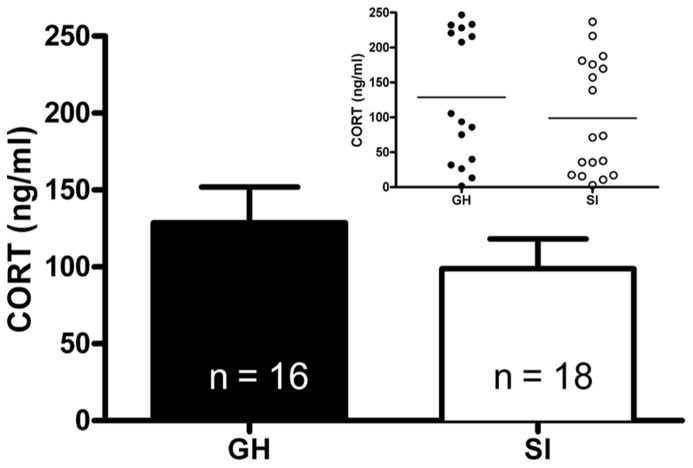
**Baseline plasma corticosterone (CORT).** At PND 85–95 (after 6 weeks of GH/SI), baseline CORT levels did not differ between group housed (GH) and socially isolated (SI) rats (*t* = 0.09867, *p* = 0.1656). The inset shows the individual differences in CORT levels within groups.

**FIGURE 4:**
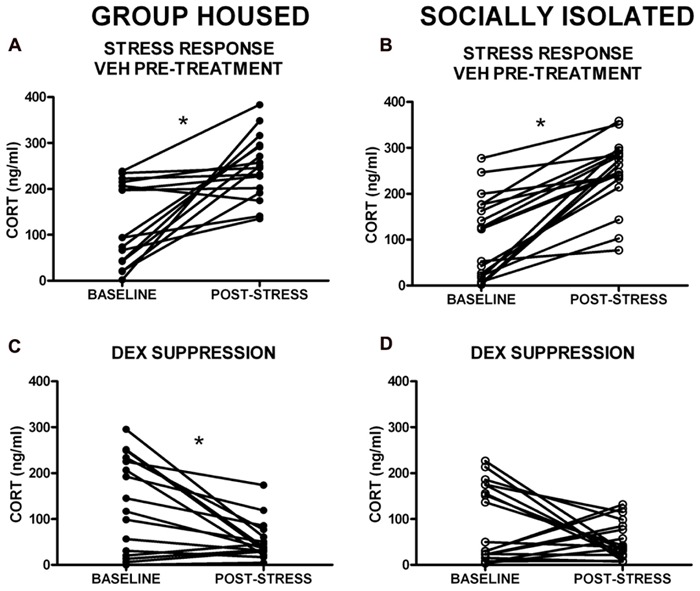
**Corticosterone (CORT) levels in response to swim stress with VEH and dexamethasone (DEX) pre-treatment.** **(A,B)**. A main effect of treatment group indicated that stress significantly elevated CORT levels (VEH pre-treatment), and **(C,D)** DEX pre-treatment resulted in a significant suppression of CORT levels. **(D)** Follow-up *t*-tests within groups showed that SI rats failed to suppress CORT in response to DEX. *p < 0.05 baseline vs. post-stress.

Given the within-subject design, counterbalanced 2-day testing procedure used for the DST, baseline CORT levels were averaged for the two testing days for comparison of baseline CORT values between groups. There was no group difference in baseline CORT levels (**Figure 3**). However, the driving hypothesis of these studies was that HPA axis function would be disrupted in SI rats (i.e., CORT level in response to a stressor and negative feedback), without requiring a difference in baseline CORT levels. To test this hypothesis, baseline CORT levels were compared to post-stress CORT levels using a two-way RM ANOVA [group (GH, SI) × treatment (VEH or DEX)]. There was a main effect of treatment [*F*(3,135) = 56.4, *p* < 0.001], but no significant interaction. Bonferroni *post hoc* tests indicated that with VEH pre-treatment, there was a significant increase in CORT (*p* < 0.05), and that with DEX pre-treatment, there was a significant suppression of CORT (*p* < 0.05; **Figures 4A–D**). Given that our a priori hypothesis was that HPA axis dysfunction would be present in SI rats, additional within-group *t*-tests were conducted to compare pre- and post-stress measures of CORT. Following DEX pre-treatment and 5 min swim stress, GH rats showed significant suppression of plasma CORT (*t* = 3.513, *p* < 0.01; **Figure 4C**), whereas SI rats failed to show significant suppression, indicating a modest impairment of negative feedback in SI rats (*t* = 1.629, *p* = 0.061; **Figure 4D**).

### ETHANOL INTAKE AND PREFERENCE

**FIGURE 5:**
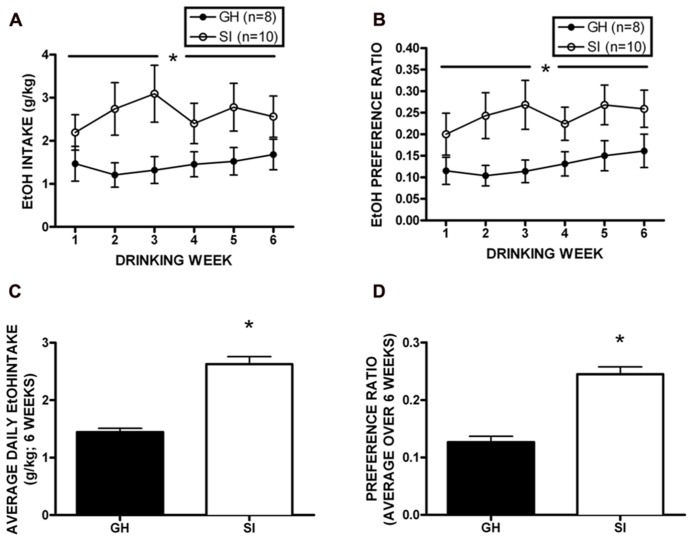
**Homecage, two-bottle choice intermittent access ethanol (EtOH) self-administration.** Socially isolated (SI) rats drank significantly more EtOH than group housed (GH) rats over the 6 week period [*F*(1,96) = 18.73, *p* < 0.01; **A**], and SI rats showed significantly greater preference for EtOH [*F*(1,95) = 21.48, *p* < 0.01; **B**]. SI rats drank nearly twice as much as GH rats (*t* = 8.165, *p* < 0.001, SI: 2.63 ± 0.13 g/kg; GH: 1.44 ± 0.08 g/kg; **C**), and showed significantly greater preference (*t* = 7.394, *p* < 0.001; SI: 0.245 ± 0.01; GH: 0.127 ± 0.01; **D**). *p < 0.05 SI vs. GH rats.

Following behavioral testing, approximately half of the cohort was single housed for the duration of the studies to test homecage, intermittent access two-bottle choice EtOH self-administration (*n* = 18). Daily drinking data were averaged across week, and a two-way RM ANOVA showed that SI rats drank significantly more EtOH than GH rats over the 6 week self-administration period [*F*(1,96) = 18.73, *p* < 0.01; **Figure 5A**], and SI rats showed significantly greater preference for EtOH [*F*(1,95) = 21.48, *p* < 0.01; **Figure 5B**]. Indeed, when intake and preference data were collapsed across the entire self-administration period, SI rats drank approximately twice as much as GH rats (*t* = 8.165, *p* < 0.001, SI: 2.63 ± 0.13 g/kg; GH: 1.44 ± 0.08g/kg; **Figure 5C**), and showed significantly greater preference (*t* = 7.394, *p* < 0.001; SI: 0.245 ± 0.01; GH: 0.127 ± 0.01; **Figure 5D**).

### RELATIONSHIP AMONG MEASURES OF ANXIETY-LIKE BEHAVIOR, ETHANOL INGESTION, AND CORTICOSTERONE

**FIGURE 6:**
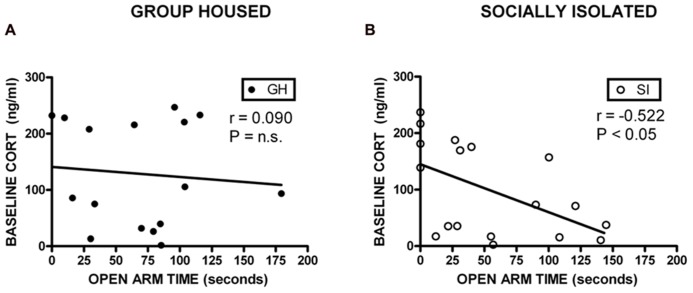
**Correlations between corticosterone (CORT) and anxiety-like behavior.** For SI rats, there was a significant negative correlation between baseline CORT and open arm time **(B)**, indicating that higher levels of baseline CORT were correlated with greater anxiety-like behavior, and lower levels of CORT were correlated with less anxiety-like behavior. No correlation was observed in GH rats **(A)**.

**FIGURE 7:**
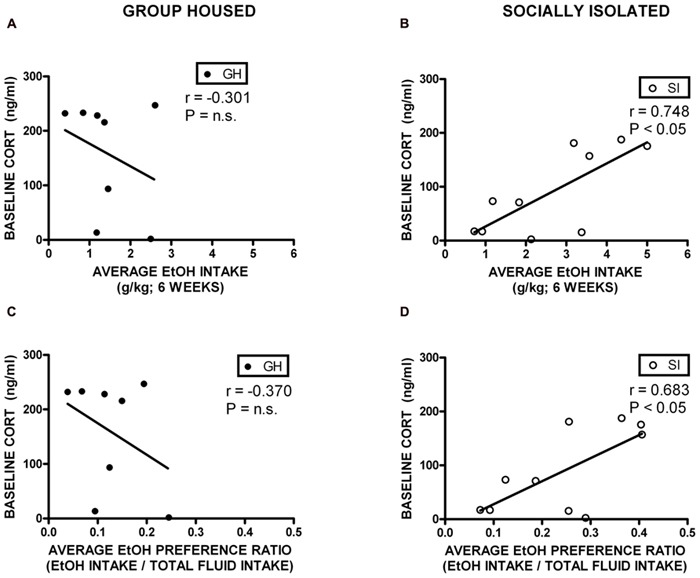
**Correlations between corticosterone (CORT) and ethanol (EtOH) intake and preference.** SI rats showed a significant positive correlation between baseline CORT and average EtOH intake **(B)** and baseline CORT and average EtOH preference **(D)**. Conversely, GH rats’ baseline CORT was not significantly correlated with average EtOH intake **(A)** or preference **(C)**.

Correlational analyses were conducted to assess potential relationships between HPA axis function and measures of anxiety-like behavior and EtOH self-administration/preference. Measures included in these analyses were baseline CORT, open arm time (anxiety-like behavior), magnitude of CORT suppression in the DST, magnitude of CORT increase with VEH pre-treatment, EtOH intake, and EtOH preference. Among GH rats, baseline CORT did not correlate with anxiety-like behavior (open arm time; *r* = -0.0902, *p* = 0.740; *r* = -0.00868, *p* = 0.975; **Figure 6A**). However, for SI rats, there was a significant negative correlation between baseline CORT and anxiety-like behavior (open arm time; *r* = -0.522, *p* < 0.05; **Figure 6B**), meaning that higher levels of baseline CORT were correlated with less time on the open arms (greater anxiety-like behavior). SI rats also showed a significant positive correlation between baseline CORT and average EtOH intake (*r* = 0.748, *p* < 0.05; **Figure 7B**) and baseline CORT and average EtOH preference (*r* = 0.683, *p* > 0.05; **Figure 7D**). Conversely, GH rats’ baseline CORT was not significantly correlated with average EtOH intake (*r* = -0.301, *p* = 0.469; **Figure 7A**) or preference (*r* = -0.370, *p* = 0.367; **Figure 7C**). For both GH and SI rats, anxiety-like behavior was not significantly correlated with the magnitude of the increase in CORT following stressor with DEX pre-treatment or VEH pre-treatment. These data suggest that greater levels of baseline CORT correlate with greater anxiety-like behavior and precede greater EtOH intake and preference in SI, but not GH, rats.

### POST-ETHANOL ANXIETY-LIKE BEHAVIOR AND HPA AXIS FUNCTION

Following the 6 week EtOH self-administration period, GH and SI rats were re-tested on the DST and the EPM. As before, there was no difference in baseline CORT between GH and SI rats (*t* = 0.5047, *p* = 0.6206; data not shown). On the DST, both GH and SI rats with VEH pre-treatment showed significantly elevated CORT levels after a 5 min swim stress (GH: *t* = 3.446, *p* < 0.01; SI: *t* = 5.563, *p* < 0.001). However, in response to DEX pre-treatment, SI rats showed significant suppression of CORT and GH rats showed a trend toward significant suppression of CORT (**Table 2**). GH and SI rats did not show differences in measures of anxiety-like behavior (**Table 3**), and there were no significant correlations between post-EtOH CORT and post-EtOH measures of anxiety-like behavior in GH or SI rats (data not shown).

**Table 2:** Group housed (GH) and socially isolated (SI) rats were re-tested using the dexamethasone (DEX) suppression test after 6 weeks of ethanol (EtOH) self-administration in a homecage, intermittent access model.

	**Group housed, GH**		**Socially isolated, SI**	
	**Baseline**	**post-stress**	**Baseline**	**post-stress**
VEH	146.0 ± 29.8	268.0 ± 14.5*	139.7 ± 14.7	223.9 ± 19.8*
DEX	145.1 ± 29.6	97.3 ± 18.1	120.9 ± 19.8	54.9 ± 11.3*

Both groups showed a significant stress-induced elevation in corticosterone (CORT) following vehicle (VEH) pre-treatment. In response to DEX pre-treatment, SI rats showed significant suppression of CORT and GH rats showed a trend toward significant suppression of CORT (*p < 0.05 vs. baseline).

**Table 3:** Group housed (GH) and socially isolated (SI) rats were re-tested on the elevated plus maze after 6 weeks of EtOH self-administration in a homecage, intermittent access two-bottle choice model.

	**Group housed, GH**	**Socially isolated, SI**	***t*, *p***
Open arm time (sec)	87.15 ± 21.7	89.20 ± 22.1	*t* = 0.065, *p* = 0.474
Open arm entries	3.75 ± 0.8	4.10 ± 1.0	*t* = 0.271, *p* = 0.395
Open arm explorations	7.25 ± 1.7	7.10 ± 1.2	*t* = 0.074, *p* = 0.471
Closed arm entries	6.50 ± 1.1	5.80 ± 0.7	*t* = 0.574, *p* = 0.287

*GH and SI rats did not differ on any measure. Data are presented as mean ± SEM.*

### GLUTAMATERGIC PLASTICITY IN THE BASOLATERAL AMYGDALA FOLLOWING SOCIAL ISOLATION/GROUP HOUSING

**FIGURE 8:**
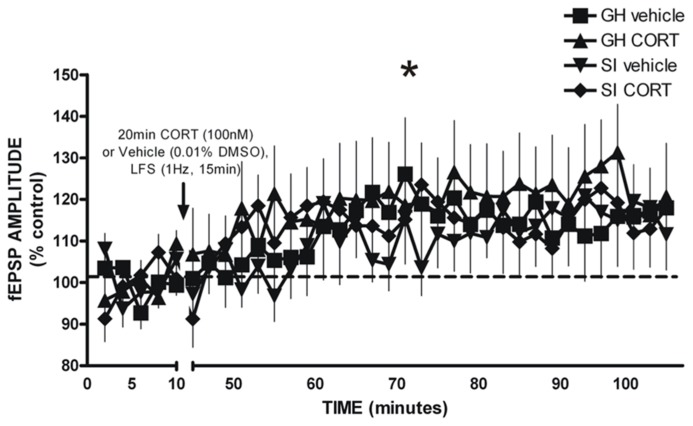
**LFS-induced long-term potentiation of glutamatergic field excitatory postsynaptic potentials (fEPSP) amplitude in the BLA of GH and SI rats.** There was a significant main effect of time, such that significant potentiation of fEPSP amplitude was observed post-LFS. *p < 0.05 post-LFS vs. baseline and drug application.

Glutamatergic fEPSPs were measured in the BLA following GH/SI and behavioral procedures to determine if plasticity was affected by GH/SI, and to determine if application of CORT would affect the induction of plasticity in either group. A three-way ANOVA (group × treatment × time) of fEPSP amplitude indicated a main effect of time [*F*(2,95) = 9.982, *p* < 0.001], such that post-LFS amplitude was significantly potentiated compared to the fEPSP response during drug application and baseline (*p* < 0.05). Thus, the LFS protocol induced plasticity that was not different between groups, nor was plasticity affected by CORT exposure. **Figure 8**.

## DISCUSSION

The current data suggest that adolescent social isolation interacts with HPA axis function, resulting in a significant correlation between greater levels of baseline plasma CORT and anxiety-like behavior in SI rats, as well as a significant correlation between higher levels of baseline CORT and greater EtOH intake and preference in SI rats. Importantly, no relationship was detected among these variables in GH rats. Together these data suggest that HPA axis dysfunction may be related to the expression of a negative affective state and vulnerability for EtOH-related behaviors following chronic early life stress, and/or that group housing imparts resilience for negative affect and an addictive phenotype.

Anxiety-like behavior was assessed using the EPM. Previous studies comparing GH and SI rats on the EPM have shown more variable outcomes using this assay relative to other measures (Zhang et al., 2012), reporting less anxiety-like behavior among SI rats (Fone et al., 1996), greater anxiety-like behavior among SI rats (Chappell et al., 2013), or no difference in anxiety-like behavior (Brenes et al., 2009; Simpson et al., 2012). Despite numerous reports from our lab and others demonstrating that the SI protocol employed in this study engenders significantly greater anxiety-like behaviors on the EPM (McCool and Chappell, 2009; Chappell et al., 2013), this current cohort showed only a non-significant trend for SI rats to exhibit greater anxiety-like behavior than GH rats. The mean time spent in the open arms for both GH and SI rats was similar to a previous study demonstrating greater anxiety-like behavior in SI rats, but with greater *n* values (McCool and Chappell, 2009). Outbred rats within the same strain have shown significant individual variability in their initial response to aversive stimuli (Wislowska-Stanek et al., 2013), suggesting that individual differences in SI rats before the housing procedure may interact with environmental stress to influence proclivity for developing anxiety-like behavior. As a group, SI rats did show significantly greater locomotion and exploratory behavior in the open field test compared to GH rats, which is one of the most consistent findings in GH/SI rodents among studies and across laboratories (Gentsch et al., 1981; Varty et al., 1999; Chappell et al., 2013).

Baseline CORT levels did not differ between groups. Previous studies measuring plasma CORT in GH/SI rats have shown no difference between groups (Weiss et al., 2004; Colaianna et al., 2013), greater CORT levels in SI rats (Serra et al., 2005) or greater CORT levels in GH rats (Raz, 2013), though these studies vary in methodology and use of different strains of rats/mice. Importantly, however, there was a correlation between anxiety-like behavior and baseline CORT in SI rats. Effects of CORT have been shown to depend on the stress/CORT state of the animal. That is, Rao et al. (2012) showed that CORT administration in rats resulted in anxiolysis if the rat was primed with an injection of CORT, but saline pre-treatment before CORT administration resulted in anxiogenesis. If the behavioral output (anxiety-like behavior) is dependent upon the current level of circulating CORT, and individual rats showed marked variability in baseline levels of circulating CORT (as in the current dataset), this would explain why SI rats with low baseline CORT showed less anxiety-like behavior, and why SI rats with high baseline CORT showed greater anxiety-like behavior. Among GH rats there was no relationship between CORT and anxiety-like behavior. Rather, CORT levels in GH rats seemed to be distributed into two distinct groups (high and low CORT), possibly engendered by their markedly more complex living environment where behavior can be greatly influenced by social rank.

The period during which rats were isolated in the current study, from immediately post-weaning through adolescence, represents a critical developmental period for social behaviors, brain function (Spear, 2000), and stress responsivity during adulthood (Romeo et al., 2006). Environmental enrichment, which can include social interaction, is associated with resilience to the expression of anxiety- and depressive-like behaviors (Konkle et al., 2010). Alternatively, several studies have highlighted that differences in the behavioral phenotype of rats are influenced by social rank (dominant vs. subordinate; Pohorecky, 2006). Social rank was not evaluated in the current study; however, an intriguing possibility for the lack of a correlation between anxiety-like behavior and CORT observed in GH rats is the possibility of two behavioral phenotypes determined by social rank. Indeed, social rank has been shown to influence open field behavior, response to EtOH, voluntary EtOH intake (Pohorecky, 2006), and the neuroendocrine response to DEX and CRH (Corticotropin-releasing hormone; Pohorecky et al., 2004). Pohorecky et al. (2004) showed that relative to dominant rats, subordinate rats have significantly elevated levels of CORT and failure to suppress CORT on a DST; in fact, GH subordinate rats are more like SI rats in terms of their neuroendocrine responses.

A central interest of the current study was the level of CORT following VEH or DEX pre-treatment, representing response to a stressor and negative feedback, respectively. In a DST, individuals with healthy (or normal) HPA axis function respond to DEX with significantly lower CORT levels relative to baseline. CORT levels were suppressed in GH rats. CORT levels were not significantly suppressed in SI rats after DEX pre-treatment, suggesting impaired negative feedback of the HPA axis in SI rats; however, it is clear from the data that a trend was present. Using a higher concentration of DEX, Serra et al. (2005) did show significantly suppressed levels of CORT after DEX pre-treatment in SI rats that was not seen with a much lower concentration (3 μg/kg), suggesting that a higher concentration of DEX may have been necessary in the current studies to see markedly impaired negative feedback in SI rats instead of the modest impairment that was observed. Alternatively, other measures of HPA axis function may have been useful in more fully characterizing HPA axis dysfunction hypothesized to occur in SI rats. For instance, in SI rats, other models have shown potentiated CORT levels in response to ACTH challenge, reduced basal ACTH (Serra et al., 2005), or significantly elevated CRH and ACTH in SI rats (Colaianna et al., 2013). Also contrary to the study hypotheses, DEX suppression was not substantially altered by 6 week EtOH self-administration as is seen in a subset of long-term alcoholics (Costa et al., 1996; Besemer et al., 2011). This is likely related to the fact that, although SI rats consumed significantly more EtOH than GH subjects, EtOH intake levels were too low to induce measures of physiological dependence.

As a group, SI rats drank twice as much EtOH as GH rats across a 6 week self-administration period. A significant positive correlation was also present between baseline CORT and EtOH intake/preference in SI rats, but not GH rats. It is possible that motivation to drink EtOH was quite different for SI and GH rats. Having always been isolated, perhaps SI rats that have higher CORT drink more to alleviate negative affect. However, for GH rats that had been living in a social group containing dominant and subordinate rats, perhaps removing subordinate rats from that environment and keeping them singly housed for the EtOH self-administration procedure was the stress-reducing event; thus, subordinate GH rats were not motivated to drink because their anxiety was alleviated by being removed from an aversive housing environment, making their intake levels similar to those of dominant rats which tend to self-administer less EtOH than subordinate rats (Blanchard et al., 1987). These data are consistent with data from a non-human primate model of developmental SI in which mean cortisol levels during 4 weeks of social separation was significantly and positively correlated with EtOH intake later in life (Fahlke et al., 2000). Fahlke et al. (2000) ran a multiple regression analysis and showed that cortisol level was the strongest predictor of adult alcohol consumption, rather than rearing condition or gender. Together, the current data and past studies suggest the importance of examining individual differences in CORT/HPA axis function in the context of chronic stress and housing environment on EtOH intake/preference.

The BLA plays an integral role in anxiety and reinforcement/addiction, and is susceptible to CORT-related plasticity. We hypothesized that glutamatergic plasticity would be occluded in SI rats and that *in vitro* CORT exposure would have differential effects in the BLA of GH and SI rats. However, group differences were not detected in the ability of the plasticity protocol to produce LTP, nor was the magnitude of LTP affected by exogenous exposure to CORT. Lack of a group difference in this measure does not preclude other changes in BLA plasticity that affect neuronal transmission and behavior. For instance, plasticity in the BLA following chronic stress *in vivo* has been shown to correlate with dendritic hypertrophy and increased spine density, which was correlated with short and long-term increases in anxiety-like behavior (Vyas et al., 2002, 2004; Vyas and Chattarji, 2004; Mitra et al., 2005). An alternative explanation that would still support the hypothesis of greater BLA excitability in SI rats may be related to GABAergic signaling. The current studies isolated glutamatergic fEPSPs by bath applying the GABA_A_ antagonist bicuculline. Recent data from our laboratory measuring GABAergic plasticity in the BLA from GH and SI rats shows striking differences in the polarity of the plasticity induced by LFS, suggesting that early life stress may primarily influence GABAergic inhibition in the BLA (Skelly et al., data not yet published). Additionally, plasma CORT levels can show significant variation from brain levels of CORT, with CORT levels remaining elevated in some brain regions long after plasma levels have returned to baseline (Little et al., 2008). Thus, the lack of a group difference in baseline CORT and small, albeit significantly different, effects on the initial DST allows the possibility that CORT affects other parameters of neural plasticity in the BLA or other brain regions that are important in negative affect/addiction.

In summary, the current studies show important relationships between baseline CORT, anxiety-like behavior, and EtOH intake/preference in rats exposed to SI during a critical developmental period. These correlations and the complexity of HPA axis function and its contribution to normal and pathological physiology and behavior begs further study in this model which engenders addiction vulnerability in SI rats, as evidenced by the markedly greater EtOH intake and preference in SI rats. Future studies must continue to characterize neurobiological correlates of behavior and the relative importance of individual differences in GH and SI rats.

## AUTHOR CONTRIBUTIONS

Tracy R. Butler and Jeffrey L. Weiner designed the experiment, analyzed the data, and prepared the manuscript. Tracy R. Butler and Olusegun J. Ariwodola conducted the experiments.

## Conflict of Interest Statement

The authors declare that the research was conducted in the absence of any commercial or financial relationships that could be construed as a potential conflict of interest.
